# Sex differences in factors associated with cannabis use among emerging adults

**DOI:** 10.3389/fpsyg.2026.1869003

**Published:** 2026-07-23

**Authors:** Sharlene D. Newman, Chuong Bui

**Affiliations:** Alabama Life Research Institute, The University of Alabama, Tuscaloosa, AL, United States

**Keywords:** ACES, alcohol, cannabis, dissociation, sex

## Abstract

Cannabis use is prevalent among young adults and is influenced by a range of psychological, behavioral, and environmental factors. Although some individuals report using cannabis to manage stress or trauma-related symptoms, the relative contribution of early adversity and concurrent psychosocial factors to cannabis use—and whether these associations differ by sex—remains unclear. This study examined the extent to which adverse childhood experiences (ACEs), depressive and anxiety symptoms, alcohol use, and dissociative experiences are associated with cannabis use in men and women. A total of 1,210 undergraduates (84% female) completed surveys assessing these domains. R-squared decomposition analyses were used to quantify each factor’s contribution to variance in cannabis use by sex. Alcohol use was the strongest predictor across both sexes. ACEs explained a greater proportion of variance among men (14.8%) than women (5.7%), with household incarceration most influential for men and household substance use most influential for women. Dissociative symptoms were uniquely associated with cannabis use among women. These findings suggest that cannabis use in emerging adults reflects multiple, sex-specific pathways involving both early life experiences and current psychosocial factors, underscoring the need for tailored prevention and intervention strategies.

## Introduction

Cannabis is the most commonly used federally illicit substance in the United States, with nearly 19% of individuals reporting past-year use (Substance Abuse and Mental Health Services Administration, 2022). Use is particularly prevalent among young adults, a developmental period characterized by increased experimentation and risk-taking behaviors. At the same time, rates of cannabis use disorder (CUD) have risen, raising concerns about the factors that contribute to both initiation and problematic use (Hasin and Walsh, 2020). Although some individuals report using cannabis to manage stress or trauma-related symptoms such as insomnia or intrusive memories (Shannon and Opila-Lehman, 2016), evidence supporting its therapeutic benefit for psychological disorders remains inconclusive (Wilson et al., 2026).

Cannabis use does not occur in isolation but is influenced by a constellation of psychological, behavioral, and environmental factors. Mental health symptoms including depression and anxiety are consistently associated with increased likelihood of cannabis use and misuse (Zarei et al., 2021; Sun et al., 2017). Co-occurring substance use, particularly alcohol consumption, is also a strong predictor of cannabis use patterns (Duke et al., 2018). Together, these factors suggest that cannabis use may function, for some individuals, as part of a broader pattern of coping with psychological distress.

Among the environmental and developmental factors associated with cannabis use, adverse childhood experiences (ACEs) represent a particularly important and well-documented risk factor. ACEs include potentially traumatic events such as physical, sexual, and emotional abuse, as well as household dysfunction due to divorce, domestic violence, parental mental illness, substance use, or incarceration (Felitti et al., 1998). The Centers for Disease Control and Prevention estimate that one in seven U. S. children experiences abuse or neglect annually (CDC, 2024). A substantial body of research links ACE exposure to increased risk of substance use and substance use disorders across the lifespan (Dube et al., 2003; Patel et al., 2025; Shahunja et al., 2025; Walsh and Cawthon, 2014).

Dissociative experiences—characterized by disruptions in perception, memory, identity, and awareness—are a common response to traumatic stress (Loewenstein, 2018; Nijenhuis and Van der Hart, 2011), particularly among individuals with histories of ACEs (Kate et al., 2020). Within the trauma literature, dissociation is often conceptualized as a coping mechanism that allows individuals to psychologically distance themselves from overwhelming or threatening experiences (Committee on Child Maltreatment Research, Policy, and Practice for the Next Decade: Phase II et al., 2014; Van der Kolk, 1994). Although this response may be adaptive during or immediately following trauma exposure, persistent or habitual dissociation can become maladaptive and interfere with emotional processing, daily functioning, and psychological well-being (Humtsoe and Deuri, 2025). Prior research has also shown that cannabis use can induce dissociative-like experiences (Ricci et al., 2021). Together, these findings suggest that individuals with histories of childhood trauma may use cannabis, in part, to achieve psychological distancing from trauma-related distress, potentially reinforcing dissociative coping processes.

Sex differences have been observed across many of the factors associated with cannabis use. Clinical and preclinical studies demonstrate sex-specific responses to cannabis (CB). For example, male cannabis users exhibit higher circulating levels of delta-9-tetrahydrocannabinol (THC), the primary psychoactive component of cannabis (Jones et al., 2012), report greater cardiovascular and subjective effects (Leatherdale et al., 2007), and are less likely to be cannabis-only users (Hasin et al., 2008). Preclinical studies further support sex-dependent effects. In animal models, male guinea pigs show greater sensitivity to the hyperphagic and hypophagic effects of CB1 receptor agonists and antagonists, respectively (Diaz et al., 2009), as well as to associated thermoregulatory changes (Farhang et al., 2009). In contrast, female rats exhibit greater catalepsy, antinociception, and locomotor effects (Tseng and Craft, 2004), along with reductions in exploratory behavior and emotionality/anxiety levels (Biscaia et al., 2003).

Sex differences are evident in both trauma exposure and subsequent psychological outcomes. Females are more likely to experience sexual abuse, whereas males more frequently experience physical abuse (Tolin and Foa, 2008; Finkelhor et al., 2009). Following trauma, males are more likely to develop externalizing responses, including substance use, while females are more likely to exhibit internalizing symptoms such as depression and anxiety (Cavanaugh et al., 2015). Biological and psychosocial differences in stress reactivity and coping may contribute to these divergent responses. Females tend to exhibit greater hypothalamic–pituitary–adrenal (HPA) axis sensitivity to interpersonal stress (Mengelkoch and Slavich, 2024), whereas males are more likely to engage in behavioral disinhibition and detachment-oriented coping (Matud, 2004; Wang et al., 2007; Perry et al., 2021). These differences may influence both the likelihood of cannabis use and the motivations underlying use, supporting the examination of sex as a moderating factor in cannabis-related risk.

Despite growing recognition of these influences, much of the existing literature has examined ACEs, mental health, and substance use in isolation rather than as part of an interconnected system of risk. In particular, limited work has quantified the relative contribution of specific ACEs, psychological symptoms, and co-occurring substance use to cannabis use, or examined how these relationships differ by sex. The present exploratory, cross-sectional study addresses this gap by examining the relative contributions of adverse childhood experiences, depressive and anxiety symptoms, alcohol use, and dissociative experiences to cannabis use in emerging adults. Using R-squared decomposition analyses, we quantify the extent to which each factor explains variance in cannabis use separately for males and females. Consistent with prior literature, we hypothesized that (1) greater exposure to ACEs and higher levels of psychological distress would be associated with increased cannabis use, and (2) the relative influence of specific ACEs and psychological factors would differ by sex, with internalizing pathways more prominent among females and externalizing pathways more prominent among males.

## Methods

### Participants

This study was conducted in accordance with the ethical principles of the Declaration of Helsinki and approved by the Institutional Review Board at the University of Alabama. All participants provided informed consent prior to participation. The sample consisted of undergraduate students enrolled in an introductory psychology course who completed study requirements for course credit.

### Measures

All measures were administered online via Qualtrics.

#### Adverse childhood experiences

The Adverse Childhood Experiences Questionnaire (Felitti et al., 1998) is a 10-item measure assessing exposure to childhood adversity (described as “While you were growing up”), including emotional, physical, and sexual abuse as well as household dysfunction (e.g., parental separation, domestic violence, household mental illness, substance use, or incarceration). Each item was scored dichotomously (0 = no, 1 = yes). Table 1 presents prevalence rates for each ACE in the current sample.

**Table 1:** Prevalence (%) of adverse childhood events (*n* = 1,210).

Items		Overall (*n* = 1,210)	Female (*n* = 1,015)	Male (*n* = 195)	*p*-value
1	Emotional abuse	19.3	19.2	19.5	0.929
2	Physical abuse	10.7	9.9	14.9	0.042
3	Sexual abuse	4.8	4.9	4.1	0.622
4	Didn’t feel loved/protected	12.6	13.2	9.7	0.183
5	Basic needs not met	3.1	2.8	4.6	0.168
6	Parent separation	28.4	28.6	27.7	0.803
7	Domestic violence	5.5	5.5	5.1	0.827
8	Household substance abuse	17.2	16.9	18.5	0.607
9	Household mental illness	17.4	18.0	14.4	0.216
10	Incarcerated household member	4.4	4.4	4.1	0.836

#### Depression

Depressive symptoms were assessed using the Patient Health Questionnaire-2 (PHQ-2; Spitzer et al, 1999), a brief screener that asks about symptoms in the past two weeks with demonstrated sensitivity (79%) and specificity (86%) for depressive disorders (Löwe et al., 2005). Items were rated on a 4-point scale (0 = not at all to 3 = nearly all the days). The score range is 0–6 with a score of 3 or higher being a positive screen.

#### Anxiety

Anxiety symptoms were measured using the Generalized Anxiety Disorder-2 (GAD-2; Spitzer et al., 2006), a two-item screener commonly used in population research that asks about symptoms in the past 2 weeks. Items were rated on a 4-point scale (0 = not at all to 3 = nearly all the days). The score range is 0–6 with a score of 3 or higher being a positive screen.

#### Alcohol use

The Alcohol Use Disorders Identification Test–Consumption (AUDIT-C; Bush et al., 1998) is a 3-item screening tool assessing alcohol consumption and hazardous drinking. Scores range from 0 to 12, with thresholds of ≥4 for men and ≥3 for women indicating possible alcohol use disorder (Bradley et al., 2007). Research has shown that problematic drinking in women is associated with lower AUDIT thresholds than in men (Geneste et al., 2012). Because the objective of this study was to identify problematic drinking, we used the established sex-specific AUDIT cutoff scores recommended in the literature (Bradley et al., 2007) rather than applying a single threshold across both groups.

#### Cannabis use

Cannabis use was assessed using the Cannabis Use Disorders Identification Test–Revised (CUDIT-R; Adamson and Sellman, 2003), an 8-item measure asking about use over the past 6 months, validated for identifying DSM-5 cannabis use disorder. Scores ≥8 indicate hazardous use, and scores ≥12 suggest possible cannabis use disorder.

#### Dissociation

The Brief Dissociative Experiences Scale–Modified (DES-B; Dalenberg and Carlson, 2010) is an 8-item measure assessing dissociative experiences over the past 7 days. Items were rated on a 5-point scale (0 = not at all to 4 = more than once a day), and average scores were used in analyses. Example questions include (*I find myself staring into space and thinking of nothing, I find that I did things that I do not remember doing.*) The total score can range from 0 to 32, with higher scores indicating greater severity of dissociative experiences. This scale has been shown to be both reliable and clinically useful in field trials. The DES family of instruments has demonstrated good reliability and validity across clinical and nonclinical populations (Dubester and Braun, 1995; Arzoumanian et al, 2023). Cronbach’s alpha was computed for the sample and was found to be good (0.8).

### Statistical analysis

The R-squared decomposition analysis was used. In a linear regression with *n* regressors, $Y=\beta_{0}+\sum_{j=1}^{n}\beta_{j}X_{j}+\varepsilon$ , the joint contribution of *X = (X_1_,…, X_n_)* in explaining *Y* can be measured by the coefficient of determination *R^2^*, defined as the explained sum of squares (SSE) over the total sum of squares (SST):

$$R^{2}=\frac{\mathrm{SSE}}{\mathrm{SST}}=\frac{\sum {\left(\hat{Y}-Y\right)}^{2}}{\sum {\left(Y-\bar{Y}\right)}^{2}}$$

Where $\bar{Y}$ is the average value of Y, and $\hat{Y}$ the predicted value of Y. Let *C_j_* denote the contribution of variable *X_j_* to the value of R^2^. Shapley-Owen-Shorrocks decomposition is a method to decompose R^2^ into individual contribution *C_j_* such that $\sum_{j=1}^{n}C_{j}=R^{2}$ . For each variable *X_j_*, *C_j_* is given by:

$$C_{j}=\sum_{k=0}^{n-1}\frac{(n-k-1)!k!}{n!}(\sum_{s\subseteq S_{k}\backslash \{X_{j}\}:|s|=k}[R_{\left(s\cup X_{j}\right)}^{2}-R_{s}^{2}])$$

Where *n* is the total number of regressors in the original regression, $S_{k}\backslash \{X_{j}\}$ is the set of all sub-models that contains *k* regressors and excludes variable *X_j_*. The proportion of R^2^ that can be attributed to *X_j_,* calculated as *C_j_ / R^2^*, can be interpreted as the relative importance (RI) of *X_j_*. Relative importance ranges between 0 and 1 (or 0 and 100%).

In the current study, the dependent variable was cannabis use (CUDIT). The independent variables included gender, PHQ-2, GAD-2, AUDIT-C, DES-B, and the items of ACE-10. In one analysis, cannabis use was regressed on 10 ACE items. Shapley decomposition was used to assess the relative importance of those ACE items. In the next analysis, PHQ-2, GAD-2, AUDIT-C, and DES-B was added to the regression model. This second analysis assessed (1) how important the ACE items were relative to the added variables, and (2) whether the ranking among the 10 ACE items changed after additional variables were included. Given that the sample was female predominant, analyses were performed for the whole sample, as well as for male and female separately.

## Results

### Sample characteristics

The final sample included 1,216 undergraduate participants, the majority of whom were women (84.0%). Participants were on average 18.6 years old (SD = 0.97), with ages ranging from 17 to 30 years (one 17 year old was excluded in the sample as one requirement was that participants were 18 years old). Three participants who identified as nonbinary were excluded from analyses due to the small sample size.

On mental health screeners, the mean PHQ-2 score was 1.30 (SD = 1.49), with 17.3% of the sample screening positive for probable depression (score ≥ 3). The mean GAD-2 score was 2.19 (SD = 1.77), with 32.2% of participants meeting the cutoff for probable anxiety disorder (score ≥ 3). Dissociation scores on the DES-B averaged 0.95 (SD = 0.66).

### Substance use

Cannabis use, as measured by the CUDIT-R, was relatively low in this sample. The mean CUDIT-R score was 1.38 (SD = 3.88). Most participants (81.5%) reported no cannabis use in the past year, while 3.0% fell into the hazardous use range (8 ≤ CUDIT-R < 12), and 3.9% met criteria consistent with possible cannabis use disorder (CUDIT-R ≥ 12).

Alcohol use was more common. The mean AUDIT-C score was 2.67 (SD = 2.80). Over one-third of participants (37.1%) reported no alcohol use, but nearly half (44.9%) scored above the threshold for possible alcohol use disorder (AUDIT-C ≥ 3 for women, ≥ 4 for men).

### Prevalence of adverse childhood experiences

Descriptive statistics for the 10 ACE items are presented in Table 1. The most commonly reported ACE was parental separation or divorce (28.5%), followed by emotional abuse (19.2%), household mental illness (17.4%), and household substance abuse (17.2%). Additional adversities included lack of feeling loved or protected (12.6%) and physical abuse (10.7%). Less common experiences were domestic violence (5.5%), sexual abuse (4.8%), parental incarceration (4.4%), and unmet basic needs (3.1%). Given the sample was female predominant, sample characteristics were provided for the whole sample as well as for each gender (Table 2).

**Table 2:** Sample characteristics.

Variable	Overall (*n* = 1,210)		Female (*n* = 1,015)		Male (*n* = 195)		*p*-value	d
	Mean	SD	Mean	SD	Mean	SD		
Age	18.63	0.96	18.57	0.82	18.93	1.48	0.001	0.38
PHQ-2	1.30	1.49	1.35	1.51	1.08	1.40	0.023	0.18
GAD-2	2.19	1.77	2.32	1.78	1.54	1.60	0.001	0.44
CUDIT	1.38	3.88	1.26	3.66	2.01	4.86	0.045	0.19
AUDIT	2.67	2.80	2.62	2.67	2.93	3.40	0.238	0.11
DES-B	0.95	0.66	0.95	0.67	0.95	0.65	0.922	0.01
	Freq	%	Freq	%	Freq	%		OR
Depression (PHQ-2 ≥ 3)	209	17.3	182	17.9	27	13.9	0.167	1.36
Anxiety (GAD-2 ≥ 3)	389	32.2	353	34.8	36	18.5	0.001	2.36
No cannabis use	986	81.5	833	82.1	153	78.5	0.033	
Normal cannabis use	139	11.5	120	11.8	19	9.7		1.16
Hazardous use (8 ≤ CUDIT < 12)	37	3.1	26	2.6	11	5.6		0.43
Cannabis use disorder (CUDIT ≥ 12).	48	3.9	36	3.6	12	6.2		0.55
No alcohol use	449	37.1	360	35.5	89	45.6	0.023	
Normal alcohol use	218	18.0	185	18.2	33	16.9		1.39
Alcohol use disorder (women, AUDIT-C ≥ 3; men, ≥ 4)	543	44.9	470	46.3	73	37.4		1.59

*p-*values were from t-tests or Chi-square tests that examine if a certain characteristic differed by gender. d = Cohen’s d. OR = female vs. male odds ratio with “No cannabis/alcohol use” being the reference category.

### Relative importance of ACEs in predicting cannabis use

**Figure 1:**
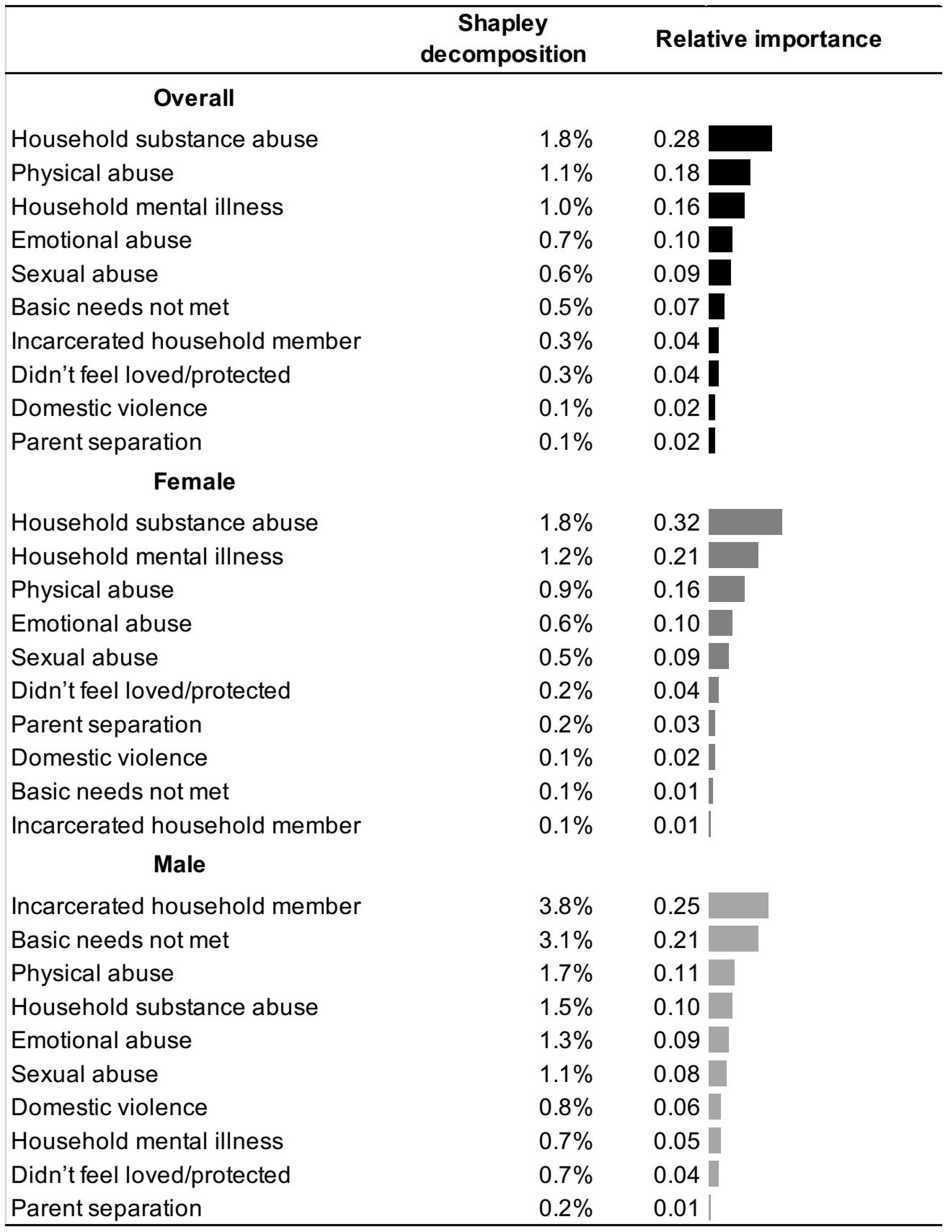
Importance of ACE-10 items in explaining in cannabis use.

Shapley decomposition analyses revealed sex differences in the predictive importance of ACEs for cannabis use (Figure 1; Table 2). Among women, the 10 ACE items together explained 5.7% of the variance in cannabis use. Household substance abuse contributed the most unique variance (relative importance = 0.32), followed by household mental illness (0.21) and physical abuse (0.16).

In contrast, among men, ACEs accounted for a larger proportion of variance (14.8%). The most influential predictors were having an incarcerated household member (0.25), unmet basic needs (0.21), and physical abuse (0.11).

### Relative importance after accounting for mental health and substance use

**Figure 2:**
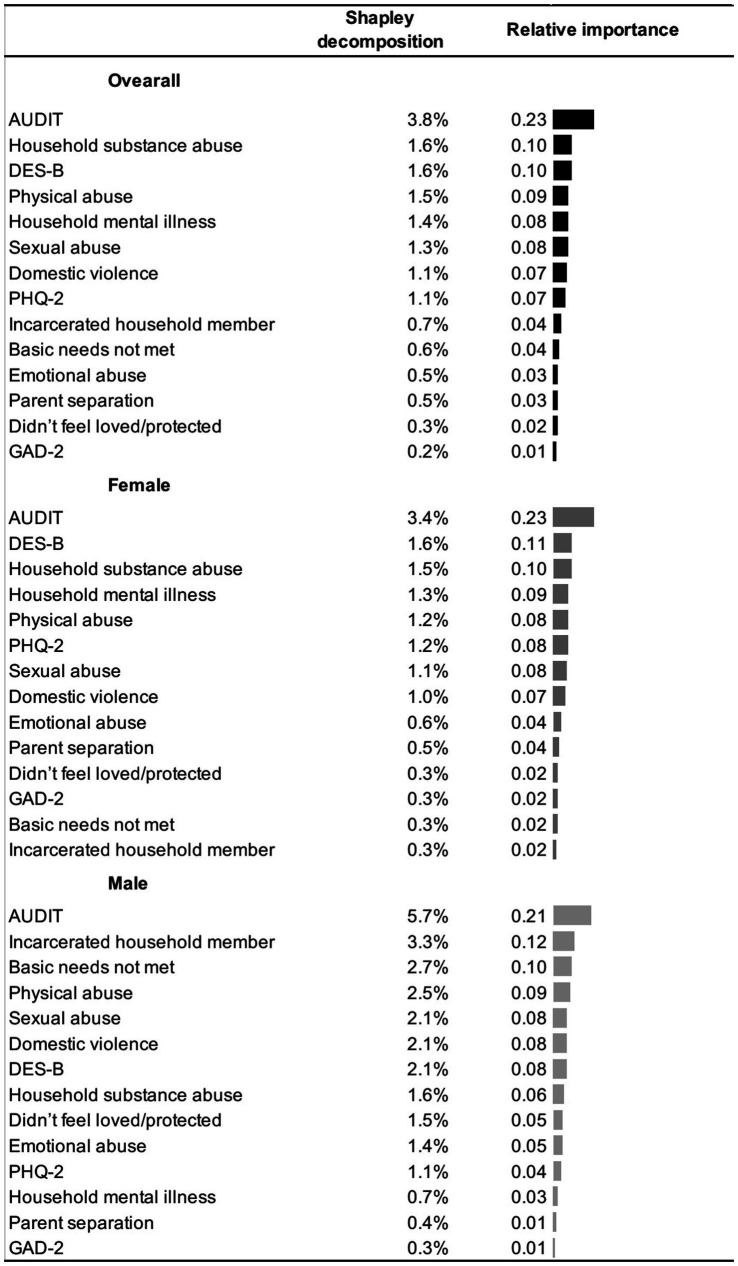
Importance of ACE-10 items, adjusting for AUDIT, PHQ-2, GAD-2, DES-B.

When additional predictors were included - depressive symptoms (PHQ-2), anxiety symptoms (GAD-2), alcohol use (AUDIT-C), and dissociation (DES-B) - the overall variance explained increased substantially for both groups (Figure 2).

For women, the expanded model explained 14.7% of the variance in cannabis use. ACEs explained for 8.2%; mental health and substance use variables together explained for 6.5%.^1^ Alcohol consumption emerged as the strongest predictor (relative importance = 0.23), followed by dissociation (0.11). Household substance abuse (0.10), household mental illness (0.09), and physical abuse (0.08) remained the top ACE-related predictors.

For men, the expanded model explained 27.5% of the variance. ACEs explained for 18.3%; mental health and substance use variables together explained for 9.2%. As with women, alcohol use was the strongest overall predictor (0.21). The ACEs with the greatest importance were parental incarceration (0.12), unmet basic needs (0.10), and physical abuse (0.09). Notably, the ranking of ACEs remained consistent before and after accounting for additional psychosocial factors, underscoring the robustness of these associations.

For both males and females, in the expanded model, PHQ-2 and GAD-2 did not make the top 5 most important predictors. GAD-2 was among the 3 least important predictors. PHQ-2 was more important in explaining CUDIT in females than in males.

## Discussion

This study examined sex-specific factors associated with cannabis use among college students, with a focus on the relative contributions of adverse childhood experiences (ACEs), mental health symptoms, alcohol use, and dissociative experiences. The findings highlight that cannabis use in emerging adults is shaped by a constellation of influences, with notable differences in the relative importance of these factors by sex. Across both men and women, alcohol use emerged as the strongest predictor of cannabis use, consistent with prior work linking polysubstance use to elevated risk for problematic cannabis use (Connor et al., 2013). In contrast, ACEs accounted for a greater proportion of variance in cannabis use among men (14.8%) than women (5.7%), suggesting that early environmental risk may play a more prominent role in shaping cannabis-related behaviors among men, while other proximal factors may be more influential among women.

Importantly, the specific predictors of cannabis use differed by sex. Among men, parental incarceration and unmet basic needs were the most influential ACE-related factors, whereas among women, parental substance use and household mental illness were more strongly associated with cannabis use. These patterns align with broader literature demonstrating sex differences in externalizing and internalizing responses to adversity (Cavanaugh et al., 2015). For example, parental incarceration has been linked to behavioral dysregulation and externalizing outcomes in males, while being more strongly associated with internalizing symptoms among females (Luk et al., 2023). Conversely, the association between parental substance use and cannabis use among women is consistent with evidence that family dysfunction and parental substance use are particularly influential on daughters’ risk for substance use (Van Den Bree et al., 2004; Kuo et al., 2021). Together, these findings suggest that cannabis use may reflect different underlying risk pathways across sexes.

The results also point to distinct psychosocial mechanisms associated with cannabis use. Dissociative symptoms were uniquely associated with cannabis use among women, suggesting a potential coping-related pathway. Dissociation is a common response to trauma (Putnam, 2009; Parfait et al., 2022) and has been conceptualized as a strategy for managing overwhelming internal experiences (Spiegel, 1963; Brenner, 1999). Given evidence that cannabis can induce dissociative-like states (Ricci et al., 2021), one interpretation is that some individuals, particularly women, may use cannabis as a means of modulating internal distress through dissociative processes. Although sex differences in dissociation remain mixed in the literature (Sar, 2011), the present findings suggest that dissociation may represent an important, and potentially modifiable, mechanism underlying cannabis use in this group. If some individuals use cannabis to induce or maintain a dissociative state that provides temporary relief from trauma-related distress, then interventions aimed at reducing dissociative coping and strengthening adaptive emotion regulation may decrease cannabis use. In this way, the underlying need to disengage from distressing internal experiences, rather than cannabis use itself, may constitute an important intervention target.

Beyond individual psychological factors, the findings are consistent with ecological models of substance use that emphasize the interaction of family, social, and psychological influences (Ennett et al., 2008). For both men and women, experiences within the family environment emerged as important predictors of cannabis use, although the specific patterns differed by sex. Among women, household substance abuse and household mental illness were among the most influential adverse childhood experiences, and dissociative symptoms remained an important predictor even after accounting for other psychosocial factors. In contrast, among men, parental incarceration and unmet basic needs consistently ranked among the strongest predictors across models. Notably, depressive and anxiety symptoms contributed relatively little unique variance in cannabis use for either sex, suggesting that broader developmental and environmental experiences may play a more prominent role than current emotional symptoms. These findings highlight that cannabis use is associated with multiple pathways of risk and that the nature of those pathways may differ for men and women.

Several limitations should be considered. First, the cross-sectional design precludes causal inference, and longitudinal research is needed to clarify the temporal relationships among ACEs, psychological symptoms, and cannabis use. Second, reliance on self-report measures introduces the possibility of recall bias and social desirability effects, particularly for sensitive experiences such as childhood adversity and substance use. Third, the predominantly female undergraduate sample limits generalizability to broader populations. However, emerging adulthood remains a critical period for studying cannabis use, as it represents both peak initiation and an important window for intervention (Scheyer et al., 2023). Another limitation is that a college population is not representative of the broader population and likely biases toward lower trauma rates. Future research should extend these findings in more diverse populations, incorporate biological and contextual moderators (e.g., stress physiology, socioeconomic conditions), and examine longitudinal trajectories to better understand the mechanisms driving cannabis use across development.

## Conclusion

Cannabis use among emerging adults is influenced by multiple interrelated factors, including early life experiences, co-occurring substance use, and psychological processes, with meaningful differences across sexes. Alcohol use was the strongest predictor across groups, while dissociation emerged as a uniquely important correlate among women. Although ACEs contributed to cannabis use, particularly among men—the findings suggest that cannabis use is best understood within a broader, multifactorial framework rather than as a direct consequence of childhood adversity alone.

The results reported in the current study highlight the importance of considering sex-specific pathways when developing prevention and intervention strategies for cannabis use. Although ACEs themselves are not modifiable, the psychological, behavioral, and social processes through which they influence cannabis use may represent important intervention targets. For example, alcohol use emerged as one of the strongest predictors of cannabis use for both men and women, suggesting that integrated approaches addressing polysubstance use may be particularly beneficial. Among women, dissociative symptoms accounted for meaningful variance in cannabis use, raising the possibility that interventions focused on trauma processing, emotion regulation, and alternative coping strategies may reduce reliance on cannabis as a means of managing distress. Among men, the importance of parental incarceration and unmet basic needs suggests that interventions addressing social and economic stressors, strengthening social support networks, and improving access to resources may be especially relevant. More broadly, the findings suggest that reducing cannabis use may require addressing the downstream consequences of childhood adversity rather than the adverse experiences themselves.

Future research should incorporate biological measures of stress and endocannabinoid functioning, as well as contextual factors such as family environment and economic conditions, to further clarify the mechanisms linking childhood adversity to cannabis use and inform tailored approaches for reducing risk among young adults.

## Data Availability

The raw data supporting the conclusions of this article will be made available by the authors, without undue reservation.

## Footnotes

1 An astute reader would notice that ACEs explains a greater amount of variance in CUDIT in the expanded model. In the expanded model, a larger R^2^ is partitioned. Hence, it is possible for the 10 ACEs to end up with a larger contribution than they do in the model without mental health and substance use variables.
